# The use of intermittent preventive treatment in pregnancy and insecticide-treated bed nets for malaria prevention by women of child-bearing age in eight districts in Malawi

**DOI:** 10.1186/s12936-015-0840-y

**Published:** 2015-08-15

**Authors:** Dyson Mwandama, Julie Gutman, Adam Wolkon, Madalitso Luka, James Jafali, Doreen Ali, Don P. Mathanga, Jacek Skarbinski

**Affiliations:** Malaria Alert Centre, University of Malawi College of Medicine, Blantyre, Malawi; Division of Parasitic Diseases and Malaria, Malaria Branch, Centers for Disease Control and Prevention, Atlanta, GA USA; National Malaria Control Programme, Ministry of Health, Lilongwe, Malawi; Malawi and Department of Community Health, College of Medicine, Lilongwe, Malawi

**Keywords:** Malaria, Intermittent preventive treatment in pregnancy, Insecticide-treated nets, Malawi

## Abstract

**Background:**

Intermittent preventive treatment in pregnancy (IPTp) and insecticide-treated bed nets (ITNs) can reduce the morbidity and mortality associated with malaria in pregnancy. Although the coverage for both IPTp and ITN use have been described in Malawi, the analysis of factors associated with IPTp receipt and ITN use is lacking. This analysis was conducted to assess IPTp and ITN use and predictors of use by women of child-bearing age (WOCBA).

**Methods:**

A two-stage cluster-sample cross-sectional survey was conducted April 16–30, 2009 in eight districts across Malawi. Information on receipt of two or more doses of IPTp, ITN ownership, and ITN use the night before the survey was collected. Multivariate logistic regression was used to assess predictors of IPTp and ITN use.

**Results:**

Data were collected from 7407 households containing 6985 WOCBA and 3213 recently pregnant women (women who reported a completed pregnancy in the 2 years before the survey). Most recently pregnant women (96 %) had at least one antenatal care (ANC) clinic visit; 91 % reported receiving at least one dose of IPTp, and 72 % reported receiving two or more doses of IPTp. Women in Phalombe, Rumphi, and Lilongwe were more likely to receive two doses of IPTp than those in Blantyre [adjusted odds ratio (aOR) 2.5 (95 % CI 1.5–4.5), 2.5 (95 % CI 1.5–4.3), and 2.0 (95 % CI 1.2–3.1), respectively]. Educated women were more likely to have received IPTp compared to women with no education [aOR 1.6 (95 % CI 1.0–2.6) for those who completed primary school, aOR1.9 (95 % CI 1.1–3.3) for some secondary school, and aOR 4.1 (95 % CI 1.9–8.7) for completed secondary school or above], and women in the poorest socioeconomic status quintile were less likely to receive IPTp than those in the least poor quintile [aOR 0.68 (95 % CI 0.48–0.97)]. In all, 53 % of WOCBA used an ITN the previous night. Women in Nkhotkhota and Phalombe were less likely to have slept under an ITN the previous night compared to those in Blantyre [aOR 0.52 (95 % CI 0.39–0.69) and aOR 0.67 (95 % CI 0.47–0.95), respectively]. In addition, age [aOR 0.61 (95 % CI 0.45–0.83) for women 15–19 years old], and either being currently pregnant [aOR 1.5 (95 % CI 1.2–2.0)] or having been pregnant in the previous 2 years [aOR 2.4, (95 % CI 2.1–2.8)] were associated with ITN use.

**Conclusion:**

In Malawi in 2009, IPTp and ITN use in WOCBA fell short of national and international goals. Adoption of new guidelines encouraging administration of IPTp at every scheduled ANC visit might increase IPTp use. Increasing health promotion activities to encourage earlier attendance at ANC clinics and create demand for IPTp and ITNs might improve overall IPTp and ITN use.

## Background

Malaria remains a major public health problem, despite tremendous efforts and substantial progress in the implementation of control programmes [1]. Malaria infection is an important risk factor for morbidity and mortality in pregnancy and particularly dangerous in primigravid women [2]. Adverse effects associated with malaria in pregnancy include maternal anaemia, intrauterine death, preterm delivery, and low birth weight (LBW) [2, 3]. However, interventions such as intermittent preventive treatment in pregnancy (IPTp) with sulfadoxine–pyrimethamine (SP) combined with insecticide-treated bed nets (ITNs) have been shown to have a protective efficacy of 21 % on the prevalence of LBW in the first or second pregnancy [4]. Therefore, the World Health Organization (WHO) currently recommends a package of interventions for control of malaria during pregnancy in areas with stable high transmission of *Plasmodium falciparum* which includes the use of IPTp, ITNs, and effective case management of malaria and anaemia [5, 6].

At the time of this study, the national policy in Malawi was in concordance with WHO recommendations and stated that all pregnant women receive at least two treatment doses of IPTp with SP, at least 4 weeks apart, and under direct observation at an antenatal care (ANC) clinic. ITN distribution in Malawi at the time of this study was via three main mechanisms: (1) routine free distribution of ITNs for children born in health facilities, children attending their first visit under the Expanded Programme on Immunization (EPI) if an ITN was not received at birth, and pregnant women at their first visit to an antenatal care (ANC) clinic; (2) periodic mass campaigns targeted at households in ‘hard to reach areas’; (3) traditional social marketing through private sector outlets [7]. Under this policy, all pregnant women were supposed to receive one free ITN at their first ANC visit, and a second at delivery, in an attempt to ensure that all pregnant women and their infants were protected. By the time of the survey, 73 % of households with either a pregnant woman or child under-5 years of age owned an ITN [8].

Although Malawi has a long history of recommending IPTp for pregnant women, and success in scaling up ITNs, however the variation by district and relative importance of different factors by district has not been conducted. In this analysis, IPTp and ITN use and predictors of use were assessed in eight districts in Malawi in order to identify the main drivers/barriers to IPTp and ITN use, and guide the programme to design appropriate interventions.

## Methods

Malaria transmission in Malawi is holo-endemic and peaks in October–April. Parasitaemia is frequent in pregnant women of all gravidities; between 1997 and 2006, 10.4 % of women delivering at Queen Elizabeth Central Hospital in Blantyre, Malawi had peripheral parasitaemia, and in Machinga in 2010, 5.3 % had peripheral parasitaemia [9, 10].

### Household survey

**Fig. 1:**
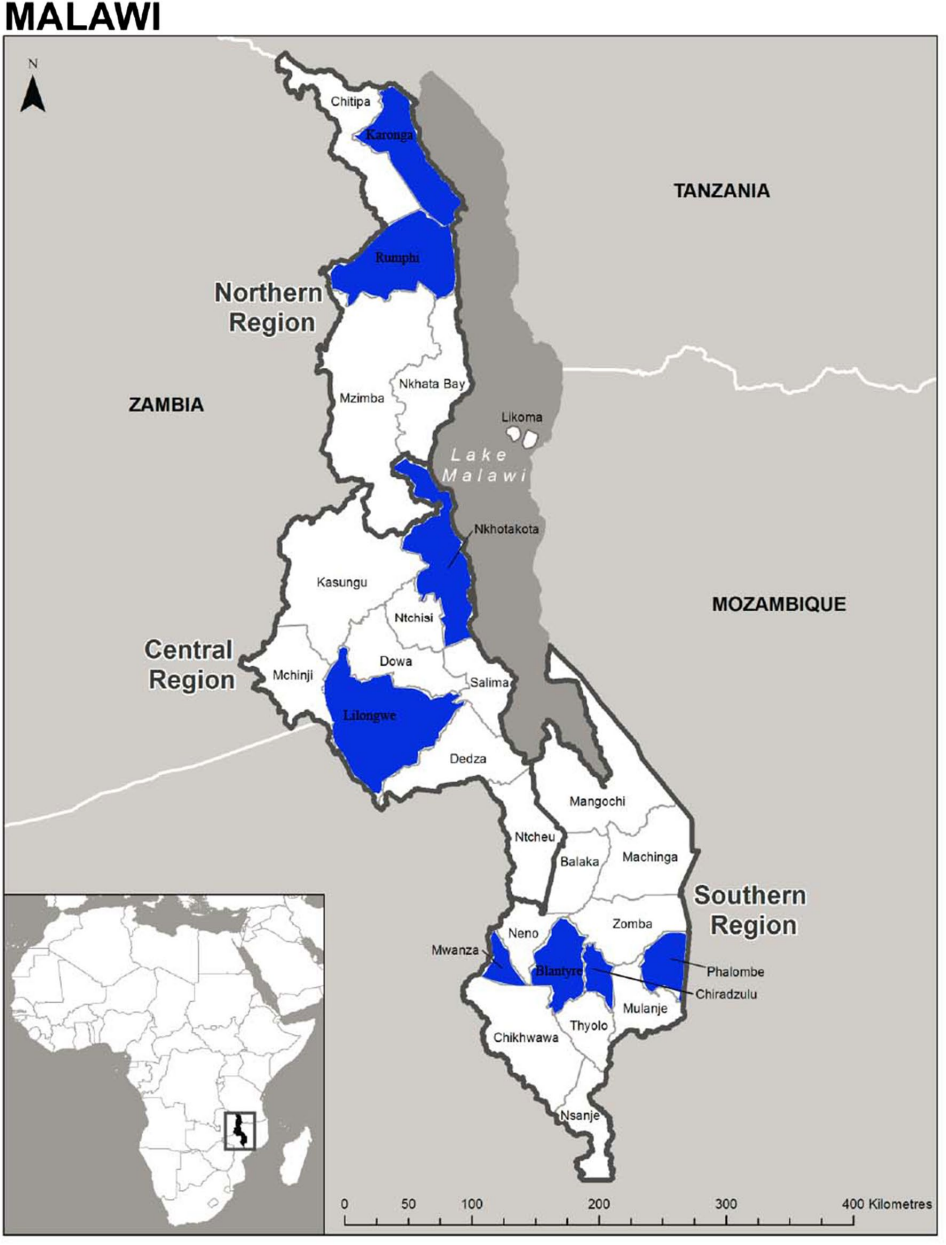
Map of Malawi showing eight survey districts

The household survey has been described in detail elsewhere [8]. Briefly, we conducted a two-stage cluster-sample cross-sectional household survey April 16–30, 2009 at the end of the long rains, in the middle of the high malaria transmission season. The study was conducted in both urban and rural communities in eight of the 28 districts in Malawi (Lilongwe, Blantyre, Mwanza, Chiradzulu, Phalombe, Rumphi, Nkhotakota, and Karonga), which were selected in collaboration with the Ministry of Health to serve as sentinel districts to monitor malaria burden and coverage of malaria control interventions—but did not constitute a nationally representative probability sample. These districts contain approximately 33 % of the entire population of Malawi and are dispersed in the north, central, and south regions (Fig. 1). A two-stage cluster sampling design was used. The first stage was composed of selecting enumeration areas (EAs). Altogether, 30 EAs per district were chosen using systematic random sampling with selection probability proportional to estimated size using the 1998 census. In the second stage, the EAs were divided into segments of roughly 30–60 households and a segment was randomly selected using a personal digital assistant (PDA; Dell Axim X50s, Dell Inc., Austin, TX, USA) with a specially-designed programme for random segment selection developed by the Centers for Disease Control and Prevention, USA. All households or a randomly selected subset of households in a selected segment were invited to participate in the survey. Informed consent was obtained from the head of household or other adult household resident. All the minors between 7 and 17 years were asked to provide an assent to participate in the study. All household members were asked to participate. A household listing was created which included all people who usually live in the household as well as guests of the household who stayed in the household the previous night. Information on the number, and visual inspection on the integrity of the bed nets in the household was collected by creating a bed net roster, which included information on whether nets were ITNs or conventional nets. Using the bed net roster and household listing, we recorded which household member slept under which bed net, allowing us to collect detailed, linked information on household members and bed nets in order to explore useful information on the ITN ownership and use. Women of child-bearing age (WOCBA) were defined as all women aged 15–49 years. The use of IPTp was defined as the uptake of at least two doses of sulfadoxine–pyrimethamine (SP) with at least one received at an ANC visit under direct observed therapy.

### Ethical approval

Ethics approval was obtained from the Malawi College of Medicine Research and Ethics Committee (COMREC) in Blantyre and Centers for Disease Control and Prevention (CDC) Institutional Review Board in Atlanta, GA, USA.

### Data analysis

Analyses were performed using SAS version 9.2 (SAS Institute, Cary, NC, USA) using the proc survey procedures, which use the Taylor expansion method to account for cluster sampling and unequal selection probabilities. Analyses were weighted and weights equaled the inverse of the exact probability of selection. Percentages reported in this report reflect this weighting unless otherwise noted. Results were defined as statistically significance if the p value was <0.05.

Multivariate logistic regression was used to assess predictors of IPTp and ITN use. The association of district, age (categorical in 5 year increments), education (categorical variable including categories for no education, some primary school, completed primary school, some secondary school, and completed secondary school or above), socioeconomic status by wealth quintile assessed by wealth asset index, and where applicable, pregnancy category (currently pregnant, completed pregnancy in past 2 years, not pregnant in past 2 years), on the outcomes of interest was assessed.

## Results

### Characteristics of WOCBA

A total of 7407 households containing 29,806 persons were surveyed. Of these, 6985 (23.4 %) were WOCBA, and 3213 of WOCBA (45.9 %) reported having completed a pregnancy in the 2 years prior to the survey. The mean age of all WOCBA was 28.1 years (95 % CI 27.8–28.3) (Table 1). Overall, 34 % of WOCBA completed a primary education or higher, but this varied by district with 18 % of WOCBA in Nkhotakhota District and up to 65 % of WOCBA in Rumphi District having completed a primary education or higher. Overall, 53 % of women reported sleeping under an ITN the night before the survey.

**Table 1:** Characteristics of women aged 15–49 years in eight districts, Malawi, 2009 (N = 6985)

	Blantyre (N = 787) % (95 % CI)	Chiradzulu (N = 822) % (95 % CI)	Karonga (N = 845) % (95 % CI)	Lilongwe (N = 874) % (95 % CI)	Mwanza (N = 1068) % (95 % CI)	Nkhotakhota (N = 878) % (95 % CI)	Phalombe (N = 784) % (95 % CI)	Rumphi (N = 927) % (95 % CI)	Total (N = 6985) % (95 % CI)
Mean age	27.2 (26.6–27.8)	28.3 (27.8–28.8)	28.2 (27.6–28.8)	28.6 (28.0–29.1)	27.4 (27.0–27.9)	28.7 (27.9–29.4)	27.8 (27.1–28.6)	27.5 (28.9–28.1)	28.1 (27.8–28.3)
Ethnicity									
Chewa	9 (6–12)	2 (0–3)	3 (1–6)	79 (66–92)	16 (7–27)	66 (53–80)	<1 (0–1)	4 (1–6)	40 (32–49)
Yao	22 (16–27)	30 (18–42)	1 (0–3)	5 (0–10)	1 (0–2)	4 (1–6)	<1 (0–1)	1 (0–2)	10 (7–13)
Tumbuka	3 (1–5)	0	34 (21–46)	4 (1–7)	<1 (0–1)	4 (1–8)	0	91 (85–97)	8 (6–10)
Lomhwe	27 (18–35)	61 (48–74)	<1 (0–1)	3 (1–4)	3 (1–5)	1 (0–1)	98 (96–100)	2 (0–3)	19 (15–23)
Other	40 (32–47)	7 (2–12)	61 (47–76)	9 (3–16)	80 (70–89)	24 (13–36)	1 (0–1)	3 (0–6)	23 (19–27)
Education									
None	6 (3–10)	19 (14–23)	4 (3–6)	21 (15–28)	17 (13–21)	16 (10–24)	23 (16–30)	7 (3–12)	16 (13–19)
Some primary school	38 (32–44)	55 (51–59)	54 (47–61)	52 (44–61)	62 (57–67)	65 (57–73)	62 (57–68)	28 (21–35)	50 (46–54)
Completed primary school	17 (14–21)	13 (10–16)	20 (17–24)	8 (5–11)	9 (5–12)	6 (3–8)	8 (5–10)	34 (28–39)	12 (10–14)
Some secondary school	22 (17–27)	11 (8–14)	16 (11–20)	12 (6–18)	10 (6–14)	10 (6–14)	5 (3–7)	20 (15–26)	14 (11–17)
Completed secondary school or above	16 (12–20)	2 (1–4)	6 (2–9)	6 (1–11)	3 (1–5)	2 (0–4)	2 (1–3)	11 (5–16)	8 (5–10)
Socioeconomic status by asset index									
Poorest	11 (7–15)	13 (10–16)	10 (5–14)	25 (18–33)	28 (23–33)	27 (20–35)	18 (13–23)	6 (4–9)	19 (16–22)
Less poor	13 (10–16)	19 (15–22)	11 (8–14)	14 (10–18)	19 (15–23)	21 (16–27)	21 (17–25)	13 (9–16)	15 (13–17)
Middle	13 (10–17)	29 (23–34)	19 (15–24)	19 (15–24)	21 (18–24)	18 (14–22)	32 (28–35)	20 (14–27)	19 (17–22)
More rich	19 (15–24)	30 (23–37)	29 (24–35)	15 (11–18)	19 (16–23)	16 (13–19)	22 (17–28)	22 (16–27)	19 (17–21)
Least poor	43 (34–53)	9 (6–13)	31 (21–40)	27 (13–41)	13 (8–18)	18 (7–28)	7 (5–9)	39 (28–49)	28 (21–34)
Used insecticide-treated bed net previous night	57 (53–62)	54 (49–59)	54 (49–60)	52 (43–60)	60 (56–65)	42 (39–46)	54 (48–60)	52 (45–58)	53 (50–57)
Used untreated bed net previous night	15 (10–20)	5 (3–6)	16 (11–21)	10 (4–15)	2 (0–4)	21 (16–26)	3 (1–5)	7 (2–13)	11 (8–13)

*95* *% CI* 95 % confidence interval

### ANC clinic and IPTp use

Among the women who reported having completed a pregnancy in the 2 years prior to the survey, ANC use was high in all districts with 96 % of women attending ANC clinic at least once during their last pregnancy (Table 2). Overall 72 % received two or more doses of IPTp with 57 % of women in Nkhotakota and up to 83 % of women in Rumphi having received two or more doses of IPTp.

**Table 2:** Use of antenatal care (ANC) clinics and intermittent preventive treatment in pregnancy (IPTp) with sulfadoxine–pyrimethamine (SP) by women aged 15–49 years who completed a pregnancy in the past 2 years in eight districts, Malawi, 2009 (N = 3213)

	Blantyre (N = 367) % (95 % CI)	Chiradzulu (N = 305) % (95 % CI)	Karonga (N = 307) % (95 % CI)	Lilongwe (N = 391) % (95 % CI)	Mwanza (N = 488) % (95 % CI)	Nkhotakhota (N = 386) % (95 % CI)	Phalombe (N = 503) % (95 % CI)	Rumphi (N = 466) % (95 % CI)	Total (N = 3213) % (95 % CI)
Any ANC clinic use	97 (95–99)	96 (93–99)	99 (98–100)	95 (92–98)	99 (98–100)	94 (91–97)	96 (94–99)	97 (95–99)	96 (95–97)
IPTp-SP doses received									
0	10 (7–14)	10 (6–13)	6 (2–9)	7 (4–11)	3 (1–5)	21 (15–26)	6 (3–9)	8 (4–13)	9 (7–10)
1	21 (14–28)	26 (19–32)	27 (21–33)	17 (11–22)	28 (23–33)	22 (17–28)	14 (9–19)	8 (5–12)	19 (16–22)
≥2	68 (61–76)	65 (58–72)	67 (60–74)	76 (70–82)	69 (64–75)	57 (50–64)	79 (73–86)	83 (77–89)	72 (69–75)

*95* *% CI* 95 % confidence interval

### Predictors of IPTp use

After adjusting for age, educational level, socioeconomic status, and use of ITNs or untreated bed nets, women from Lilongwe [adjusted odds ratio (aOR) 2.0, 95 % confidence interval (CI) 1.2–3.1], Phalombe (aOR 2.5, 95 % CI 1.5–4.4), and Rumphi (aOR 2.5,95 % CI 1.5–4.3) Districts were more likely to receive two or more doses of IPTp than women in Blantyre District (Table 3). Women who had completed primary school (aOR 1.6, 95 % CI 1.0–2.6), some secondary school (aOR 1.9, 95 % CI 1.1–3.3), and secondary school or above (aOR 4.1, 95 % CI 1.9–8.7) were more likely to receive two or more doses of IPTp than women who had no education. Women in the poorest wealth quintile were significantly less likely to receive two or more doses of IPTp than women in the least poor quintile (aOR 0.68, 95 % CI 0.48–0.97). Use of either an ITN (aOR 1.2, 95 % CI 0.97–1.60) or untreated bed net (aOR 1.4, 95 % CI 0.85–2.4) the night before the survey was not associated with receiving two or more doses of IPTp.

**Table 3:** Factors associated with receipt of two or more doses of intermittent preventive treatment in pregnancy (IPTp) with sulfadoxine–pyrimethamine (SP) by women aged 15–49 years who completed a pregnancy in the past 2 years in eight districts, Malawi, 2009 (N = 3213)

Variable	Unadjusted odds ratio (95 % CI)	p value	Adjusted odds ratio (95 % CI)	p value
District				
Blantyre	Referent	Referent	Referent	Referent
Chiradzulu	0.85 (0.55–1.3)	0.48	1.1 (0.67–1.7)	0.75
Karonga	0.95 (0.60–1.5)	0.83	1.0 (0.61–1.6)	0.98
Lilongwe	1.4 (0.91–2.3)	0.12	2.0 (1.2–3.1)	0.004
Mwanza	1.0 (0.69–1.6)	0.84	1.4 (0.88–2.2)	0.16
Nkhotakhota	0.60 (0.39–0.93)	0.02	0.78 (0.48–1.3)	0.31
Phalombe	1.8 (1.1–3.0)	0.02	2.5 (1.5–4.4)	<0.001
Rumphi	2.3 (1.3–3.9)	0.002	2.5 (1.5–4.3)	<0.001
Age category				
15–19 years old	1.1 (0.67–1.8)	0.70	1.1 (0.64–1.8)	0.82
20–24 years old	1.1 (0.73–1.6)	0.68	1.0 (0.65–1.5)	0.91
25–29 years old	1.6 (1.0–2.4)	0.04	1.3 (0.89–2.0)	0.16
30–34 years old	1.4 (0.93–2.0)	0.11	1.3 (0.85–1.9)	0.26
35–49 years old	Referent	Referent	Referent	Referent
Education				
None	Referent	Referent	Referent	Referent
Some primary school	1.3 (0.92–1.9)	0.13	1.4 (1.0–2.0)	0.07
Completed primary school	1.4 (0.88–2.4)	0.15	1.6 (1.0–2.6)	0.05
Some secondary school	1.7 (0.99–2.9)	0.05	1.9 (1.1–3.3)	0.03
Completed secondary school or above	4.2 (2.1–8.5)	<0.001	4.1 (1.9–8.7)	<0.001
Socioeconomic status by asset index				
Poorest	0.50 (0.34–0.74)	<0.001	0.68 (0.48–0.97)	0.03
Less poor	0.59 (0.40–0.88)	0.01	0.81 (0.55–1.2)	0.29
Middle	0.59 (0.40–0.86)	0.006	0.74 (0.52–1.1)	0.11
More rich	0.72 (0.48–1.1)	0.11	0.90 (0.61–1.3)	0.57
Least poor	Referent	Referent	Referent	Referent
Used insecticide treated bed net previous night	1.2 (0.97–1.6)	0.08	1.2 (0.97–1.6)	0.09
Used untreated bed net previous night	1.6 (0.88–2.8)	0.13	1.4 (0.85–2.4)	0.18

*95* *% CI* 95 % confidence interval

### Predictors of ITN use

In multivariate models adjusting for district, age, education level, socioeconomic status, and pregnancy status, women who resided in Nkhotakhota (aOR 0.52,95 % CI 0.39–0.69) and Phalombe (aOR 0.67, 95 % CI 0.47–0.95) Districts were significantly less likely to use an ITN the previous night than women who resided in Blantyre District (Table 4). Moreover, women aged 15–19 years (aOR 0.61, 95 % CI 0.45–0.83) were significantly less likely to use an ITN than women aged 35–49 years. Neither education level nor socioeconomic status were significantly associated with ITN use. Women who were currently pregnant (aOR 1.5, 95 % CI 1.2–2.0) or who had completed a pregnancy in the past 2 years (aOR 2.4, 95 % CI 2.1–2.8) were significantly more likely to use an ITN compared to women who had not been pregnant in the past 2 years.

**Table 4:** Factors associated with use of an insecticide treated bed net the previous night by women aged 15–49 years in eight districts, Malawi, 2009 (N = 6985)

Variable	Unadjusted odds ratio (95 % CI)	p value	Adjusted odds ratio (95 % CI)	p value
District				
Blantyre	Referent	Referent	Referent	Referent
Chiradzulu	0.87 (0.66–1.1)	0.32	0.86 (0.61–1.2)	0.37
Karonga	0.88 (0.68–1.2)	0.36	0.90 (0.68–1.2)	0.48
Lilongwe	0.79 (0.55–1.1)	0.20	0.77 (0.51–1.2)	0.22
Mwanza	1.1 (0.88–1.5)	0.33	1.1 (0.8–1.5)	0.44
Nkhotakhota	0.54 (0.43–0.68)	<0.001	0.52 (0.39–0.69)	<0.001
Phalombe	0.86 (0.65–1.2)	0.32	0.67 (0.47–0.95)	0.02
Rumphi	0.79 (0.59–1.1)	0.14	0.76 (0.55–1.1)	0.10
Age category				
15–19 years old	0.68 (0.51–0.90)	0.008	0.61 (0.45–0.83)	0.002
20–24 years old	1.2 (0.96–1.5)	0.12	0.87 (0.69–1.1)	0.21
25–29 years old	1.3 (1.0–1.5)	0.02	0.98 (0.79–1.2)	0.83
30–34 years old	1.2 (0.91–1.5)	0.22	0.94 (0.73–1.2)	0.65
35–49 years old	Referent	Referent	Referent	Referent
Education				
None	Referent	Referent	Referent	Referent
Some primary school	1.1 (0.85–1.5)	0.41	1.1 (0.85–1.5)	0.40
Completed primary school	1.1 (0.79–1.6)	0.57	1.1 (0.75–1.5)	0.69
Some secondary school	0.97 (0.66–1.4)	0.86	1.1 (0.75–1.7)	0.58
Completed secondary school or above	0.86 (0.52–1.4)	0.55	0.87 (0.52–1.5)	0.60
Socioeconomic status by asset index				
Poorest	1.1 (0.77–1.7)	0.52	1.0 (0.70–1.5)	0.93
Less poor	1.1 (0.76–1.6)	0.62	0.96 (0.69–1.3)	0.81
Middle	1.4 (0.93–2.0)	0.10	1.2 (0.87–1.7)	0.24
More rich	1.4 (1.0–2.0)	0.04	1.3 (1.0–1.8)	0.05
Least poor	Referent	Referent	Referent	Referent
Pregnancy category				
Currently pregnant	1.5 (1.2–1.9)	<0.001	1.5 (1.2–2.0)	<0.001
Completed pregnancy in past 2 years^a^	2.4 (2.1–2.8)	<0.001	2.4 (2.1–2.8)	<0.001
Not pregnant in past 2 years	Referent	Referent	Referent	Referent

*95* *% CI* 95 % confidence interval

^a^Not currently pregnant

## Discussion

IPTp and ITN use are key for prevention of malaria in pregnancy, but data from this analysis show that coverage of both IPTp and ITN use were below the 80 % national and international targets in 2009 [11], despite high levels of ANC attendance. Moreover, ITN and IPTp use varied by district suggesting sub-national disparities in coverage within Malawi. Lastly, different characteristics were significantly associated with IPTp use (e.g. education level and socioeconomic status) compared to ITN use (e.g. age and pregnancy status).

Although Malawi has higher two dose IPTp coverage than many other sub-Saharan countries, the coverage of receipt of two or more IPTp doses is low according to this survey (72 %) as well as the national Malaria Indicator Surveys (MISs) with 60 % in 2010 and 54 % in 2012 [12, 13]; all are both well below the 80 % coverage target set by the National Malaria Control Programme [12]. Since IPTp should be administered as directly observed therapy, high levels of IPTp coverage should be attainable, given high level of at least one visit to the ANC clinic observed in this study. Some possible explanations for the discrepancy between any ANC clinic attendance and low coverage of two or more IPTp doses could be due to inadequate ANC clinic attendance by all pregnant women (e.g. attendance only once or at times that IPTp could not be administered, such as after 36 weeks of gestation when IPTp-SP administration was not recommended according to Malawian guidelines at the time) or missed opportunities to administer IPTp-SP, either as a result of SP stock-outs or health worker confusion on when to give IPTp [14]. Unfortunately, data on the number of ANC clinic visits nor the timing of the visits were not collected; however, this type of data should be collected in future surveys to better understand low coverage of two doses of IPTp. Additional training of healthcare workers and improving community awareness on the importance of early attendance to ANC clinic and taking IPTp may help to improve coverage levels. WHO recently released updated guidance calling for IPTp to be administered at each ANC visit starting as early as possible in the second trimester and continuing until delivery, provided the doses are given at least 4 weeks apart [5, 15]. At the time this study was conducted, the IPTp guidelines in Malawi prohibited administration of IPTp-SP after 36 weeks gestational age. In addition, similar to the situation in other sub-Saharan African countries [16], there were discrepancies in the guidelines from Reproductive Health and the National Malaria Control Programme, which has been shown to cause health worker confusion [14]. Adoption and promotion of the revised simplified guidelines recommended by WHO should improve coverage, as this provides an opportunity to harmonize the messages, which is expected to decrease confusion and promote health worker compliance. Furthermore, removing the restriction on delivering IPTp after 36 weeks will allow more time for the last dose to be given.

ITN use in this survey was uniformly below national and international targets, with only 53 % of women using an ITN the previous night similar to the coverage level in the 2010 national MIS (51 %) [12]. The low uptake and use of ITNs has been described in many malaria endemic communities [11, 17]. In the past, the high costs of buying and re-treating ITNs with insecticide were the major barriers to high uptake [18]. For this reason, free net distribution programmes at ANC sites in Malawi were begun. In this survey, as noted previously, reported ITN use was higher among currently and recently pregnant women than among women without a recent pregnancy due distribution of ITNs at ANC clinics [8]. A similar effect was noted in Nigeria, where ITN use was 87.3 % among pregnant women given free ITNs in the ANC clinics [19]. Women under 20 years of age were less likely to use ITNs than any other age group, possibly due to the fact that this group of women was the least likely to have had a prior pregnancy and was, therefore, the least likely to have received free ITN via ANC clinic or Expanded Programme on Immunization (EPI) distribution outlets. Children aged 5–14 are the group least likely to use an ITN [20], suggesting that adolescent girls are not being prioritized to sleep under a net. As this group is at highest risk for the adverse events of malaria in pregnancy, this is a critical issue to address. Malawi’s current strategy for malaria control includes universal ITN coverage. In mid-2012, the Ministry of Health conducted a mass distribution campaign in which over six million ITNs were distributed, with a goal of covering all households with one ITN for every two people [8]. Following this campaign, ITN use among pregnant women rose to 62 % [21].

Although increasing education and socioeconomic status were associated with the use of two or more doses of IPTp, these were not associated with ITN use. The lack of noted disparity in ITN use by educational level and socioeconomic status in this study compared to previous studies might be related to free ANC-based distribution of ITNs to pregnant women [19]. As most women who were recently pregnant attended at least one ANC clinic visit, they likely had the opportunity to receive a free ITN; free ITN distribution via ANC clinics and EPI visits has been shown previously to be a major driver of ITN ownership and use in Malawi [8, 22]. As ITN use by communities as well as by individual women improves birth outcome in malaria endemic regions of Africa [23], more effort into national and community-based education is required to ensure that all WOCBA sleep under an ITN every night. This is important to help prevent infections early in pregnancy, as these infections are particularly harmful [24]. Overall access and use of ITNs has improved following the mass campaign in 2012, with 62 % of pregnant women reporting having slept under an ITN in the 2014 MIS [21], however, additional attention is needed to push coverage to the 90 % coverage target set by the NMCP for 2016 [21]. These data suggest that behaviour change communication needs to be targeted to younger women, particularly as young primigravidae are at highest risk for complications related to malaria in pregnancy. Although ITN distribution at first ANC clinic is a critical component of reducing malaria in pregnancy, ideally, woman should be sleeping under an ITN even before they become pregnant, and control programmes need to ensure that ITN distribution channels and behaviour change messages target all women to ensure both high access to ITNs and use.

Moreover, differences in IPTp and ITN use were noted by district; IPTp use ranged from 57 to 85 % and ITN use ranged from 42 to 60 %. District-level differences in coverage of both of these interventions likely reflect population-level differences (e.g. educational level and socioeconomic status) in factors that are associated with intervention use as well as differences in health system capacity to deliver these interventions (e.g. quality of ANC clinic and ITN distribution programme). The drivers of these district-level differences need to be better understood, as these differences remained even after adjusting for differences in individual-level factors (e.g. educational level and socioeconomic status). These data could be used to better target resources to districts with lower intervention coverage, such as improved health management interventions or additional resources for materials, supplies and service delivery.

### Limitations

Although the areas surveyed are geographically diverse, and contain 33 % of the population of Malawi, due to the purposive rather than probability-based selection of the 8 districts, the findings are not intended to be generalizable to all of Malawi. In addition, recall and social desirability bias may have influenced the responses to survey questions. However, where possible, responses to survey questions (i.e. ownership of assets including ITN) were confirmed by visual inspection. In addition, the survey team reviewed the ANC card to verify the receipt of SP during pregnancy. Where the ANC card was not available, interviewers probed to see if the woman took three big, white tablets at the health facility (indicative of SP). This survey did not assess the number of ANC visits made by each woman, nor the timing of these visits, inhibiting our ability to assess whether low IPTp uptake was influenced by inadequate ANC attendance.

## Conclusions

Use of two or more doses of IPTp and ITNs by women in Malawi in 2009 fell short of international and national goals. Increasing demand and coverage levels of both of these critical interventions will require a concerted and integrated effort on the part of the National Malaria Control Programme and the Reproductive Health Unit. Although ITN use has improved following mass distribution campaigns, IPTp coverage has been relatively stagnant. WHO has recently released revised guidelines recommending that IPTp be administered at every scheduled ANC clinic visit after the first trimester; it is hoped that adoption of these guidelines will result in increased uptake of IPTp, through simplifying the message delivered to healthcare workers, assuming adequate stocks of drugs are available at ANC clinics. In addition, promotional activities to encourage earlier attendance at ANC clinics and use of IPTp and ITNs should target communities and young women in particular to increase demand for, and correct use of, IPTp and ITNs.

## Abbreviations

aOR: Adjusted odds ratio
ANC: Antenatal care
EPI: Expanded Programme on Immunization
IPTp: Intermittent preventive treatment in pregnancy
ITN: Insecticide treated bed nets
LBW: Low birth weight
MIS: Malaria Indicator Surveys
SP: Sulfadoxine–pyrimethamine
WOCBA: Women of child bearing age
WHO: World Health Organization
